# Prevalence of severe mental distress and its correlates in a population-based study in rural south-west Uganda

**DOI:** 10.1186/1471-244X-11-97

**Published:** 2011-06-08

**Authors:** Eugene Kinyanda, Laban Waswa, Kathy Baisley, Dermot Maher

**Affiliations:** 1 Medical Research Council/Uganda Virus Research Institute (MRC/UVRI) Uganda Research Unit on AIDS, Entebbe, Uganda; 2 Department of Epidemiology and Population Health, London School of Hygiene and Tropical Medicine, London, UK

## Abstract

**Background:**

The problem of severe mental distress (SMD) in sub-Saharan Africa is difficult to investigate given that a substantial proportion of patients with SMD never access formal health care.

This study set out to investigate SMD and it's associated factors in a rural population-based cohort in south-west Uganda.

**Methods:**

6,663 respondents aged 13 years and above in a general population cohort in southwestern Uganda were screened for probable SMD and possible associated factors.

**Results:**

0.9% screened positive for probable SMD. The factors significantly associated with SMD included older age, male sex, low socio-economic status, being a current smoker, having multiple or no sexual partners in the past year, reported epilepsy and consulting a traditional healer.

**Conclusion:**

SMD in this study was associated with both socio-demographic and behavioural factors. The association between SMD and high risk sexual behaviour calls for the integration of HIV prevention in mental health care programmes in high HIV prevalence settings.

## Background

Governments in sub-Saharan Africa including those in Uganda, Liberia and Southern Sudan are slowly realizing that mental illness makes a significant contribution to the overall health burden which is projected to rise. Governments have therefore started to include mental health in the minimum health care package to be delivered through an integrated approach in the existing primary health care system [1-3]. Mental illness encompasses a broad range of conditions of varying degrees of severity. Severe mental distress (SMD) for purposes of this paper refers to all mental and neurological problems that are associated with severe disturbance in behaviour, thought or speech as seen in a sub-Saharan African socio-cultural setting. The underlying philosophy behind the use of this term was the need to capture all forms of severe psychological disturbances as seen at community level in an sub-Saharan African setting, where in the majority, the communities still believe that these illnesses are due to non-medical causes. We also desired to use a category amendable to use by non-medical interviewers.

SMD as conceived in this study and in this socio-cultural setting may be due to the following causes: i) severe mental illnesses- schizophrenia, paranoid psychoses and manic-depressive disorder; ii) acute transient psychoses secondary to socio-cultural stress such as the 'brain fag syndrome'; iii) psychoses resulting from cerebral involvement in infectious diseases such as malaria, typhoid fever, and HIV infection; iv) epilepsy largely due to inadequate care at child birth, malnutrition, malaria, parasitic diseases and head trauma; v) post-traumatic stress disorders secondary to conflict and civil strife, which is endemic on the continent; vi) conversion-dissociative states including mass hysteria; and vii) alcohol and marijuana use and other drug-related problems [4-9]. To assess SMD in this study we used a composite question derived from the four screening questions for 'probable psychosis' in the WHO Self Report Questionnaire-25 [10]. In a study in urban Ethiopia having at least two of the four WHO Self Report Questionnaire-25 [10] items was taken as indicative of 'probable psychosis' (for purposes of this study taken to be equivalent to SMD) where a prevalence of 5% SMD was obtained [11].

Currently in most countries in sub-Saharan Africa patients with SMD usually visit traditional healers (often the only source of mental health care sought) before seeking treatment in formal health care system largely because the predominant community attitudes to mental illness is that it is a spiritual rather than a medical problem [4,12,13]. Overall, more than three quarters of patients with severe mental illness in developing countries do not receive treatment from the formal health system [14]. However with increased health education of communities and the increased availability of mental health services through integration into general health care, this situation is set to change.

To better plan for this trend, there is an urgent need for research into the problem of severe mental distress (SMD) in sub-Saharan African settings. To study this problem in its entirety requires population-based rather than hospital facility-based studies so as to include those persons who are still seeking mental health care from traditional healers.

There have been few population-based surveys of SMD in rural communities in Africa. A large population-based cohort in rural southwest Uganda, initially established in 1989 for HIV surveillance, provided the opportunity to assess community prevalence of SMD in adults.

## Methods

### Setting

Since 1986 Uganda has been recovering from decades of previous civil, political and economic turmoil. The estimated 30 million population are mostly engaged in subsistence agriculture. Annual Gross National Income is $300 per capita and mean life expectancy at birth is 50 years [15]. It is one of the countries in Africa where the HIV epidemic was first reported and that was initially most badly affected by HIV. The country has few psychiatrists (30 in number), fewer psychologist and psychiatric social workers with most of these confined to the capital city of Kampala and a few other urban centres. Outside the capital city of Kampala, formal mental health care is mainly provided by approximately 150 psychiatric clinical officers (physician assistants with a diploma in psychiatry) and psychiatric nurses [2,3,16].

The mental health services in the country are arranged around a primary health care model built on the integration of mental health care into a decentralized health-care delivery system at district level, through a system of Health Centres (HC) starting at village level, HC-I; subcounty level, HC-II; county level, HC-III; subdistrict level, HC-IV and district level, HC-V. This health structure is supported by a referral system which includes regional referral hospitals (which have a mental health unit, that should be staffed by a psychiatrist) and the national tertiary referral hospital at Butabika located in the capital city of Kampala [2,3,17]. Traditional beliefs about mental illness are still very strong in the country with many patients with mental illnesses first seeking care from traditional healers and in many cases this is the only mental health care that they will access [12].

### Study site

The cohort comprises approximately 20,000 residents residents who live in 25 neighbouring villages in southwest Uganda a few kilometers from Lake Victoria. The vast majority of dwellings are distributed throughout the countryside rather than clustered in villages, which mainly represent administrative areas demarcated on maps rather than population centres. The study population are mostly subsistence farmers, whose staple diet consists of matooke (cooking bananas) with groundnuts. There are no tarmac roads and access may be difficult during the rains. People live in semi-permanent structures built from locally available materials. The community is stable and homogeneous, with most people from the Baganda tribe, and 15% of Rwandese origin, who are well assimilated. Religious affiliation is mostly Christian, with a significant Muslim minority (28%). Levels of literacy are low and the main income-earning activities are growing bananas, coffee and beans, and trading fish [18]. HIV seroprevalence reported in this study is a representation of the national picture [19]. HIV prevalence in the study area declined from 8.5% in 1990 to 6.2% in 1999/2000 but thereafter rose to 7.7% in 2004/2005 [20].

### Annual cohort survey

Since 1989 information has been collected in the annual cohort survey on HIV sero-prevalence and associated social, demographic and behavioural factors. Full details of the cohort and annual HIV serosurvey have been published elsewhere [21,22]. In brief, an annual household survey has been conducted since 1989, with all study village residents eligible for inclusion. Average annual serosurvey participation is about 60%-65%, although a much higher percentage has ever participated. Community sensitization activities precede each survey round, including local council briefings and village meetings. All households are visited by, in turn, the mapping, census and survey teams. Consenting residents are interviewed at home in the local language by trained survey staff and provide a blood sample for HIV testing. Since 2009, the cohort has also served as a platform for epidemiological studies on a range of other health problems of public health importance.

### Measurement of SMD and other variables

In the 20^th ^annual survey round (December 2008 - November 2009), data were collected on the prevalence of selected non-communicable diseases (including diabetes, other cardiovascular disease risk factors, and SMD) in consenting adults (defined for the purpose of the survey as aged 13 years and above) [23]. Probable SMD was assessed using a composite screening question, 'Is there a time(s) when you experienced disturbances in your behaviour, thoughts or speech, e.g. shouting, undressing, running aimlessly, hearing voices of people who were not there?' This question was derived from the four screening questions for 'probable psychosis' as is given in the WHO Self Report Questionnaire-25 [10]. Study participants who responded positively to this question were advised to attend their nearest local health facility or the study clinic for further assessment. Additional questionnaire information collected included: sociodemographic factors; socioeconomic status (SES) measured using an asset index created by combining data on 22 household possessions using principal component analysis; whether a current or past regular cigarette smoker (including both manufactured and local cigarettes); ever consulted a traditional healer; and sexual behavior. Clinical and laboratory assessments were undertaken for: diabetes (plasma glucose measured using the enzymatic reference method with hexokinase; Roche COBAS Integra 400 analyser with Glucose HK Gen.3 reagent) [24]; hypertension [25]; HIV serostatus and epilepsy (possible epilepsy was assessed using the WHO-SRQ-25 derived question, 'do you have an illness characterized by recurrent episodes of falling to the ground associated with loss of consciousness ?') [10].

### Statistical methods

Data were double-entered and verified in Access. Stata 10 (Stata Corporation, College Station, USA) was used for analyses. Age-standardised prevalence of reported SMD was calculated by combining observed prevalence with age-stratified population estimates from the 2008 census round.

**Figure 1:**
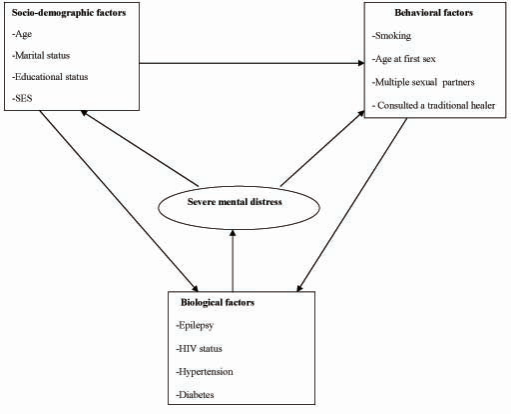
**Conceptual framework for the analysis**.

Factors associated with the observed prevalence of reported SMD were investigated using random effects logistic regression to account for correlation within households. A conceptual framework was used to consider potential determinants of and sequel to SMD (Figure 1), with factors classified into three groups: sociodemographic factors, behavioural and biological factors. A final explanatory multivariable model was not derived, since this was a cross-sectional study and it is not possible to establish causality, and many factors, such as marital breakdown, may be both possible determinants of, and sequel to, reported SMD. Instead, the association of factors of interest with reported SMD was examined, after adjusting for age and sex. These two variables were adjusted for as potentially important *a priori *confounders.

### Ethics

The study was approved by the Science and Ethics Committee of the Uganda Virus Research Institute and by the Uganda National Council for Science and Technology.

## Results

**Figure 2:**
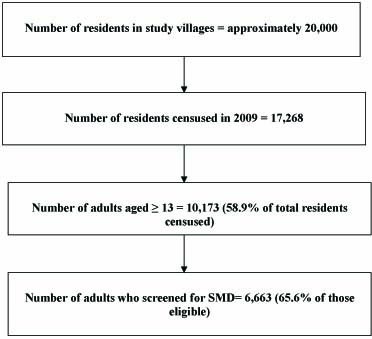
**Numbers of residents of study villages, adults censused and survey participants**.

At census, there were 4,801 males and 5,372 females aged 13 years and older resident in the study area and eligible as survey participants. Of those, 2,719 (56.6%) males and 3,959 (73.7%) females responded to the survey questionnaire (Figure 2). There was strong evidence of higher SES among non-responders, with 25.0% of non-responders in the highest socio-economic status (SES) quintile compared with 22.6% of responders (p = 0.001). There was no evidence of a difference in HIV serostatus between responders and non-responders, with 6.0% versus 6.2% being HIV seropositive, respectively (p = 0.78). Participation was lower in younger age groups, for both sexes, and among women 60 years and older.

### Characteristics of study respondents

The majority (68.5%) of respondents were under 40 years of age. About half (55.7%) had less than 7 years of formal education and 42.8% were currently married. A fifth (20.5%) of males, but only 1.3% of females, were current or past regular cigarette smokers. Three quarters (75.3%) of females and 63.2% of the males were sexually active. A small minortiy (4.2% of males and 6.0% of females) had ever consulted a traditional healer.

On clinical factors, 0.6% of males and 0.3% of females reported they were suffering from epileptic illness. HIV positive serostatus was 4.8% in males and 6.9% in females. The prevalence of probable diabetes was 0.4% in both males and females, and of probable hyperglycaemia was 3.0% in males and 2.8% in females. Of the patients with severe mental distress (SMD) none of the males and only three of the females (5.7% of those with SMD) reported that they were receiving formal medical treatment for the SMD, so majority of people who report SMD are not getting treatment.

### Prevalence of probable severe mental distress and associated factors

The observed prevalence of probable severe mental distress (SMD) (see Table 1) was 0.9% (95%CI = 0.6-1.1%), and was higher in males (1.1%, CI = 0.7-1.5) than in females (0.7%, 95%CI = 0.5-1.0%).

**Table 1:** Description of study participants responding to the question on severe mental distress (SMD)

	**Males (N = 2,719)**	**Females (N = 3,959)**
**SOCIO-DEMOGRAPHIC/ECONOMIC FACTORS^1^**		
**Age (years)**		
< 20	1037 (38.1%)	1123 (28.4%)
20-39	878 (32.3%)	1538 (38.9%)
40-59	521 (19.2%)	877 (22.2%)
≥ 60	283 (10.4%)	421 (10.6%)
**Currently married**		
Yes	1052 (38.7%)	1806 (45.6%)
**Ever married**		
Yes	1310 (48.2%)	2671 (67.5%)
**Education level**		
Less than primary	167 (6.2%)	493 (12.5%)
Incomplete primary	1398 (51.5%)	1663 (42.0%)
Primary	501 (18.4%)	851 (21.5%)
Secondary or above	651 (24.0%)	949 (24.0%)
**SES score quintile**		
Highest	560 (20.9%)	919 (23.6%)
High	607 (22.7%)	856 (22.0%)
Middle	517 (19.3%)	851 (21.9%)
Low	532 (19.9%)	781 (20.1%)
Lowest	458 (17.1%)	483 (12.4%)
**BEHAVIOURAL FACTORS^2^**		
**Current regular smoker**		
Yes	373 (13.7%)	34 (0.9%)
**Ever regular smoker**		
Yes	547 (20.2%)	50 (1.3%)
**Age at first sex**		
< 15	117 (4.3%)	343 (8.7%)
15-16	317 (11.7%)	979 (24.8%)
17-18	521 (19.2%)	996 (25.2%)
19+	492 (18.1%)	354 (9.0%)
Does not remember	271 (10.0%)	305 (7.7%)
Never had sex	1000 (36.8%)	978 (24.7%)
**Partners in past year (among sexually active)**		
None	328 (19.1%)	849 (28.5%)
1	987 (57.6%)	2073 (69.6%)
2+	398 (23.2%)	55 (1.8%)
**Consult traditional healer**		
Yes	113 (4.2%)	235 (6.0%)
**CLINICAL INDICATORS^3^**		
**Reported severe mental distress (SMD)**		
Yes	29 (1.1%)	28 (0.7%)
**Receiving treatment for SMD**		
Yes	0 (-)	3 (10.7%)
**Reported epilepsy**		
Yes	15 (0.6%)	10 (0.3%)
**HIV serostatus**		
Positive	128 (4.8%)	269 (6.9%)

^1 ^Missing marital status for 1 male and 1 female. Missing data on education for 2 males and 3 females. Missing SES index for 45 males and 69 females

^2 ^Missing data on current smoking for 2 males, and on ever smoking for 8 males and 3 females. Missing data on age at first sex for 1 male and 4 females. Missing data on partners in past year for 6 males and 4 females. Missing data on consulting a traditional healer for 13 males and 19 females.

^3 ^Missing data on reported epilepsy for 4 males and 3 females Missing data on reported SMD for 5 males and 10 females. Missing HIV status for 33 males and 43 females

In the unadjusted analysis, socio-demographic factors with the strongest association with severe mental distress were increasing age and decreasing socio-economic status (Table 2). Participants who were 40-59 years old had nearly 4 times the odds of SMD as those under 20 years of age; however, the odds of SMD in those over 60 was similar to that in those under 20. Those who were in the lowest socio-economic quintile had 3 times the odds of SMD as those in the highest socio-economic quintile. There was some evidence that females were less likely to have SMD than males (age-adjusted OR = 0.58, CI = 0.33-1.01, p = 0.05). After adjusting for age and sex, SES was the only sociodemographic factor that remained associated with SMD.

**Table 2:** Factors associated with reported severe mental distress among males and females ≥ 13 years old

	**All participants (N = 6663)**		
	
	**no. with reported disturbance/total (%)**	**Unadjusted odds ratio [95% CI]**	**Adjusted odds ratio^1 ^[95% CI]**
**SOCIO-DEMOGRAPHIC/ECONOMIC FACTORS**			
**Age (years)**			
		***P = 0.002***	***P < 0.001***
< 20	11/2155 (0.5%)	1	1
20-39	19/2410 (0.8%)	1.58 [0.73,3.41]	1.70 [0.78,3.70]
40-59	24/1396 (1.7%)	3.65 [1.72,7.74]	3.94 [1.84,8.48]
≥ 60	3/702 (0.4%)	0.85 [0.23,3.13]	0.88 [0.24,3.29]
**Sex**		***P = 0.10***	***P = 0.05***
Male	29/2714 (1.1%)	1	1
Female	28/3949 (0.7%)	0.63 [0.37,1.09]	0.58 [0.33,1.01]
**Ever married**		***P = 0.09***	***P = 0.87***
Yes	40/3971 (1.0%)	1	1
No	17/2690 (0.6%)	0.61 [0.34,1.09]	1.08 [0.43,2.74]
**Education level**		***P = 0.76***	***P = 0.77***
Secondary/above	12/1597 (0.8%)	1	1
Primary	14/1348 (1.0%)	1.39 [0.62,3.09]	1.30 [0.57,2.94]
Some primary	27/3056 (0.9%)	1.16 [0.57,2.35]	1.21 [0.59,2.51]
Less than primary	4/657 (0.6%)	0.81 [0.25,2.59]	0.76 [0.22,2.59]
**SES score quintile**		***P = 0.005***	***P = 0.009***
Highest	10/1476 (0.7%)	1	1
High	10/1457 (0.7%)	1.00 [0.39,2.54]	0.98 [0.38,2.53]
Middle	13/1366 (1.0%)	1.37 [0.57,3.32]	1.36 [0.55,3.36]
Low	5/1311 (0.4%)	0.55 [0.18,1.68]	0.52 [0.17,1.65]
Lowest	18/940 (1.9%)	3.10 [1.34,7.18]	2.90 [1.21,6.91]
**BEHAVIOURAL FACTORS**			
**Current regular smoker**		***P = 0.008***	***P = 0.13***
No	48/6254 (0.8%)	1	1
Yes	9/407 (2.2%)	3.18 [1.46,6.93]	2.03 [0.84,4.91]
**Age at first sex**		***P = 0.30***	***P > 0.99***
< 15	5/458 (1.1%)	1	1
15-16	13/1295 (1.0%)	0.91 [0.31,2.69]	0.91 [0.29,2.83]
17-18	15/1510 (1.0%)	0.91 [0.31,2.65]	0.82 [0.27,2.52]
19+	11/845 (1.3%)	1.22 [0.40,3.73]	0.93 [0.28,3.06]
Never had sex	11/1974 (0.6%)	0.50 [0.16,1.51]	0.97 [0.21,4.51]
**Partners in past year**		***P = 0.03***	***P = 0.04***
None	15/1172 (1.3%)	1.81 [0.92,3.57]	2.00 [0.96,4.16]
1	22/3055 (0.7%)	1	1
2+	9/452 (2.0%)	2.86 [1.27,6.43]	2.62 [1.06,6.44]
**Consult traditional healer**		***P = 0.001***	***P = 0.002***
No	47/6285 (0.7%)	1	1
Yes	10/346 (2.9%)	4.08 [1.94,8.58]	3.74 [1.72,8.15]
**BIOLOGICAL FACTORS**			
**Reported epilepsy**		***P < 0.001***	***P < 0.001***
No	49/6634 (0.7%)	1	1
Yes	8/25 (32.0%)	147.10 [31.95,677.16]	158.75 [31.46,801.0]
**HIV serostatus**		***P = 0.76***	***P = 0.91***
Negative	52/6191 (0.8%)	1	1
Positive	4/396 (1.0%)	1.19 [0.41,3.44]	0.94 [0.31,2.82]
**Systolic BP ≥ 140 mmHg or diastolic ≥ 90**		***P = 0.61***	***P = 0.49***
No	45/5106 (0.9%)	1	1
Yes	11/1488 (0.7%)	0.84 [0.42,1.66]	0.78 [0.38,1.59]
**Random plasma glucose ≥ 7 mmol/L**		***P = 0.10***	***P = 0.20***
No	45/5778 (0.8%)	1	1
Yes	4/194 (2.1%)	2.71 [0.93,7.83]	2.19 [0.73,6.60]

^1^Adjusted for age group and sex

In the unadjusted analysis, behavioural factors significantly associated with SMD were: being a regular smoker (OR 3.2, CI = 1.46-6.93, p = 0.008); number of sexual partners in the last year (those with none, or with two or more, sexual partners were more likely to report SMD than those with only one); and ever consulted a traditional healer (OR 4.1, CI = 1.94-8.58, p = 0.001). There was no evidence that age at first sexual encounter was associated with SMD After adjusting for age and sex, the significant association between the number of sexual partners in the last year, and consulting a traditional healer, with SMD remained, and there was weak evidence of an association with smoking.

Of biological factors, there was an extremely strong association between reported epilepsy and SMD, a relationship which remained significant even after adjusting for age and gender (P < 0.001). There was no evidence of an independent association with HIV serostatus, hypertension or diabetes with SMD in this study.

## Discussion

In this study in a poor rural community in southwest Uganda, the prevalence of probable SMD was 0.9%, similar to the 0.9% prevalence of moderate to serious mental disorders reported in Nigeria [14], but much lower than the 5% prevalence reported in urban Ethiopia using a precursor of the screening tool used in this study [11].

This study provides important new information on the community prevalence of SMD in a community rural Africa. There are two main limitations. Firstly, the use of one screening question for SMD runs the risk of assessing for a highly non-specific entity. Secondly, this question item has never been previously validated in this study population. The formation of the composite screening item was largely driven by the following considerations: the need for a question item that could be used by lay interviews; and the need to limit the number of questionnaire items in the survey. This question was however derived from an established WHO questionnaire (WHO-SRQ-25 tool) which has been used extensively in sub-Saharan Africa [10,11].

Secondly, the results of this study show that this question had criterion validity. There is however a need to validate this question item which may have utility as screening tool for severe mental distress in the context of large population-based studies where: non-mental health professionals are used in data collection; where mental health may be competing with other health disciplines for space on study questionnaires. Secondly, because this was a cross-sectional study, it was not possible to tell the direction of association between SMD and the investigated factors.

The socio-demographic factors found to be associated with SMD in this study were increasing age and low socio-economic status. A study in Ethiopia reported a significantly increased risk of mental distress with increasing age and with indices of low socio-economic status [11]. Two explanations have been offered for the association between the severe mental illness of schizophrenia and low socio-economic status. In the social causation theory, the socio-environmental factors associated with low socio-economic status (including more life events stressors, increased exposure to environmental and occupational hazards and infectious agents, poorer prenatal care and fewer support resources if stress does occur) are a cause of schizophrenia [26]. The social selection, or drift, theory is that socio-economic status is a consequence of the disorder -the insidious onset of schizophrenia is believed to preclude elevating one's status or to cause a downward drift in status [26].

On the behavioural factors, being a current smoker, having no or multiple sexual partners and having ever consulted a traditional healer were associated with SMD. After adjusting for age and sex there was a twofold increased odds of SMD among current regular cigarette smokers as compared to those who were not regular smokers. Both Lasser and colleagues (2010) and van Os and Kapur (2009) have reported higher rates of cigarette smoking among persons with mental illness as compared to population controls [27,28]. It has been suggested that patients with the severe mental illness of schizophrenia use nicotine to help reduce cognitive deficits, negative symptoms or the neuroleptic side effects [27]. The observed association between SMD and having multiple sexual partners in this study confirms what has previously been reported by other authors [29]. The association between SMD and high risk sexual behavior has been attributed to factors associated with SMD such as cognitive processing difficulties, lack of planning, and poor social skills which place these patients at risk [29]. The association between SMD and no sexual partners may reflect the severe social dysfunction associated with the severer end of the spectrum of SMD. The association between SMD and having previous contact with a traditional healer can be regarded as a form of health-seeking behavior for mental illness, a health seeking behavior that is in agreement with the predominant spiritual explanatory model for mental illness [4,13,14]. As has been reported before in low income settings, only three patients (5.7%) with SMD were receiving formal health care for their problem in this study [14].

On clinical factors, self-reported epilepsy was the only factor significantly associated with SMD. The strong association between self-reported epilepsy and SDM could be explained in two ways. Firstly, the confusional state commonly associated with the post-ictal phase of generalized seizures may lead to behavioural disturbances and hence epilepsy could be regarded as a cause of SMD. Secondly, the community may not have been able to adequately differentiate between epilepsy and SMD because of the possible overlap between SMD and epilepsy.

## Conclusion

In conclusion, this study provides a preliminary insight into the problem of SMD in a rural community in sub-Saharan Africa. The association between SMD and high risk sexual behavior in high prevalence countries such as those in sub-Saharan Africa calls for an urgent need for targeted HIV prevention measures among persons with severe mental illness. The current practice in many sub-Saharan African countries where epilepsy is mostly managed by mental health services and the observation in this study of a strong association between SMD and epilepsy; in a socio-cultural context where neurologists and psychiatrists will continue to be a rarity for the foreseeable future provides additional impetus for the need to integrate both mental health and neurological services into primary health care. Further research is under way using the cohort to investigate the associated problems of major depressive disorder and alcohol abuse/dependency.

## List of abbreviations

EK: Eugene Kinyanda; LW: Laban Waswa; KB: Kathy Baisley; DM: Dermot Maher; SMD: severe mental distress; WHO: World Health Organisation; HC: Health Centre.

## Competing interests

The authors declare that they have no competing interests.

## Authors' contributions

The authors of this manuscript made the following contributions to this manuscript Concept: EK, DM; Data collection: LW, DM; Data analysis: LW, KB, EK, DM; First draft: EK, LW, KB, DM; Final revision: EK, LW, KB, DM; Read and approved final manuscript: EK, LW, KB, DM.

## Pre-publication history

The pre-publication history for this paper can be accessed here:

http://www.biomedcentral.com/1471-244X/11/97/prepub
